# A Computational Model of Auditory Chirp-Velocity Sensitivity and Amplitude-Modulation Tuning in Inferior Colliculus Neurons

**DOI:** 10.21203/rs.3.rs-4450943/v1

**Published:** 2024-06-04

**Authors:** Paul W. Mitchell, Laurel H. Carney

**Affiliations:** 1* Department of Biomedical Engineering, University of Rochester, 601 Elmwood Ave, Rochester, NY, 14642, USA; 2* Department of Neuroscience, University of Rochester, 601 Elmwood Ave, Rochester, NY, 14642, USA

**Keywords:** Auditory midbrain, coincidence detectors, neural models, octopus cell, frequency-modulation sweeps

## Abstract

We demonstrate a model of chirp-velocity sensitivity in the inferior colliculus (IC) that retains the tuning to amplitude modulation (AM) that was established in earlier models. The mechanism of velocity sensitivity is sequence detection by octopus cells of the posteroventral cochlear nucleus, which have been proposed in physiological studies to respond preferentially to the order of arrival of cross-frequency inputs of different amplitudes. Model architecture is based on coincidence detection of a combination of excitatory and inhibitory inputs. Chirp-sensitivity of the IC output is largely controlled by the strength and timing of the chirp-sensitive octopus-cell inhibitory input. AM tuning is controlled by inhibition and excitation that are tuned to the same frequency. We present several example neurons that demonstrate the feasibility of the model in simulating realistic chirp-sensitivity and AM tuning for a wide range of characteristic frequencies. Additionally, we explore the systematic impact of varying parameters on model responses. The proposed model can be used to assess the contribution of IC chirp-velocity sensitivity to responses to complex sounds, such as speech.

## 1 Introduction

Natural sound stimuli, such as speech and music, are rich with spectral and temporal features to which auditory neurons are sensitive. The inferior colliculus (IC) has strong rate tuning for complex sound features, such as amplitude modulations (AM). Recently, physiological studies have revealed that IC neurons have diverse sensitivity to the velocity of fast frequency sweeps, known as chirps, in both periodic (Steenken et al., 2022; Henry et al., 2023) and aperiodic (Mitchell et al., 2023) stimuli. The velocity of these chirps is much greater than that of more commonly considered sounds, such as formant transitions (Liberman and Mattingly, 1989). The majority of IC neurons are sensitive for chirp velocity, regardless of characteristic frequency (CF) or MTF type (Mitchell et al., 2023). Computational models of the IC currently do not include chirp-velocity sensitivity. Velocity sensitivity arising in octopus cells of the cochlear nucleus (CN) (Lu et al., 2022), which inhibit the IC via the ventral nucleus of the lateral lemniscus (VNLL) (Adams, 1997; Vater et al., 1997), could potentially give rise to velocity sensitivity in the IC. Here, a computational model was used to test the hypothesis that a midbrain model with an inhibitory input from a velocity-sensitive octopus-cell can model the velocity sensitivity observed in IC neurons in addition to AM tuning.

Octopus cells are uniquely found in the posteroventral cochlear nucleus (PVCN) (Golding et al., 1999). These cells are excellent coincidence detectors with fine temporal resolution (Golding et al., 1995), entraining to individual periodic stimulus cycles up to 800 Hz while responding only to the onset of pure tones (Godfrey et al., 1975; Rhode and Smith, 1986). Additionally, octopus cells have a broad range of CFs (0.77–20 kHz, Godfrey et al., 1975) and are distinguished by their wide dendritic fields, which extend across a range of auditory-nerve (AN) input frequencies (Osen, 1969). Frequency-dependent dendritic delays have been suggested to counteract latencies arising from the cochlear traveling wave and to thus improve coincidence detection in response to transient stimuli (Spencer et al., 2012). Sensitivity to the timing of cross-frequency inputs with different amplitudes may also give rise to diverse sensitivity to chirp velocity (Lu et al., 2022), which is similar to that observed in IC neurons. Finally, octopus cells are known to project to the contralateral VNLL, which in turn provides an inhibitory input to the IC (Adams, 1997; Vater et al., 1997). This fact, together with the broad range of responses of octopus cells to chirp velocities, makes them a potential source of chirp sensitivity in the IC.

Lu et al. (2022) posited that octopus cells function as sequence detectors, responding preferentially to dendritic inputs with different amplitudes that arrive in a certain temporal sequence. This mechanism depends on low-voltage-activated potassium (KL) channels, which are abundant in octopus cells (Bal and Oertel, 2001). Due to the slow recovery dynamics of the KL channels, dendritic inputs evoking both subthreshold and suprathreshold excitatory post-synaptic potentials (EPSPs) are followed by relatively long periods of hyperpolarization, preventing subsequent inputs from triggering action potentials. A suprathreshold input that normally evokes an action potential on its own will not do so when preceded by a subthreshold input. Therefore, a frequency sweep that triggers the suprathreshold EPSP before the subthreshold one will result in an action potential, whereas one that triggers the subthreshold EPSP before the suprathreshold one will not. This dependence upon the temporal sequence of inputs with different amplitudes, which are presumed to be tuned to different frequencies, was proposed to explain selectivity of octopus cells for chirp direction (Lu et al. 2022).

The modeling strategy used in this paper is rooted in work by Siebert (1965), who introduced a quantitative strategy to predict psychophysical performance as a function of stimulus parameters, using an analytical model for auditory-nerve responses. This approach used statistical decision theory to define the limits of auditory discrimination based on a statistical description of neural responses. An important assumption for this approach is to treat neural responses as nonhomogeneous Poisson processes (NHPPs) (Rieke et al., 1997). Siebert’s method has been employed for discrimination of tone frequency, level, and binaural cues, such as interaural time and level differences (Siebert, 1970; Colburn, 1973; Heinz et al., 2001a,b). This strategy was further developed by Krips and Furst (2009a,b), who demonstrated that Siebert’s method can be extended into the central nervous system. Krips and Furst’s (2009b) model cells are coincidence detectors (CDs) that receive multiple inputs and respond depending upon the relative timing of excitatory and/or inhibitory inputs. This general CD-based strategy is promising for modeling both the octopus cell’s sequence-detection mechanism and, subsequently, the chirp sensitivity of IC neurons.

Here, we propose a model of IC chirp-sensitivity based on sequence detection of inhibitory octopus cells. First, we outline the model architecture within the Krips and Furst framework, describing the octopus-cell stage and then the IC stage. Then, we demonstrate the feasibility of the model in simulating IC neurons with physiologically plausible chirp sensitivity as well as AM tuning. Finally, we describe the parameters of the model and explore how parameter choice affected the sensitivity of the model cell. This model is a step towards addressing a gap in current computational models, which do not simulate sensitivity to chirps, despite its prevalence among IC neurons—over 90% are sensitive to chirp direction (Mitchell et al., 2023). In complex, harmonic sounds such as speech and music, phase differences between components give rise to chirps. Thus, increasing the accuracy of computational models will improve model predictions of responses to these realistic, perceptually important sounds.

## 2 Methods

Many modeling approaches exist for both octopus cells and IC neurons, including Hodgkin-Huxley models (octopus cells: Spencer et al., 2012; Manis and Campagnola, 2018; Lu et al., 2022; IC neurons: Cai et al., 1998), conductance-based models (octopus cells: Kalluri and Delgutte, 2003; Spencer et al., 2018; IC neurons: Hewitt and Meddis, 1994), and simpler phenomenological models (octopus cells: Rebhan and Liebold, 2021; IC neurons: Nelson and Carney, 2004). The model proposed here is based on work that extends statistical decision theory (Siebert, 1965, 1970; Colburn, 1973; Heinz et al., 2001a,b, 2002) to the central nervous system by generalizing auditory neurons as coincidence detectors that receive excitatory and/or inhibitory inputs (Krips and Furst, 2009a,b). This approach was selected for its flexibility, relatively low number of parameters, and ability to assign parameters to physiological correlates.

### 2.1 Stimuli

To validate the IC-model response characteristics, the following set of stimuli, similar to those used in the experimental study of Mitchell et al. (2023), were presented to model cells. Responses to pure tones at different levels and frequencies were used to generate response maps (RMs) and assess frequency tuning. Sinusoidally amplitude-modulated (SAM) noise was used to generate modulation transfer functions (MTFs), used to evaluate tuning to modulation frequency. Aperiodic chirp stimuli were used to generate rate-velocity functions (RVFs), which characterize sensitivity to direction and velocity of chirps. Additionally, click-train stimuli were used as an alternate method of generating MTFs, for the purpose of comparing octopus-cell model responses to physiological responses (Godfrey et al., 1975). Unless stated otherwise, all model response rates were determined by calculating the integral of the model rate function over the stimulus duration. Rate functions were constructed using the mean of responses to five stimulus repetitions. The input signal for each repetition was the mean of 10 statistically independent high-spontaneous-rate (HSR) AN-model responses for each frequency channel. The number of AN fibers per channel was chosen to align with the approximate number of HSR AN fibers that innervate each inner hair cell in the cochlea (Keithley and Schreiber, 1987).

RMs were produced using a series of 200-ms-duration tones with frequencies ranging from 250 Hz – 10 kHz, at 10, 30, 50, and 70 dB SPL. Tones had 10-ms raised-cosine ramps. Average response rates were plotted to assess CF, the frequency at which the cell was excited above spontaneous rate at the lowest sound level.

Responses to SAM noise were used to generate rate MTFs. Noise was 100% modulated over a range of modulation frequencies from 2 – 500 Hz. The noiseband spanned 100 Hz – 10 kHz, had a spectrum level of 30 dB SPL (overall level of 70 dB SPL), and duration of 1000 ms (including 50-ms raised-cosine ramps). MTFs were classified based on the rates in response to modulated relative to unmodulated stimuli. Here, the model was designed to produce band-enhanced MTF shapes, which are characterized by increased excitation, with respect to unmodulated responses, over a band of modulation frequencies (Kim et al., 2020)

An aperiodic chirp stimulus, introduced in Mitchell et al. (2023), was designed to characterize neural sensitivity to direction and velocity of fast frequency chirps using RVFs, defined as average rate versus the velocity of a linear frequency sweep. This stimulus is derived from the Schroeder-phase harmonic complex (Schroeder, 1970). To construct the aperiodic chirp stimulus, fundamental periods were extracted from a set of Schroeder-phase stimuli, with each period being equivalent to a linear frequency chirp. The set of chirp velocities used was identical to those in Mitchell et al. (2023): ±0.40, ±0.80, ±1.59, ±3.16, ±6.24, and ±9.24 kHz/ms. A random sequence of chirps was generated, with each combination of direction and velocity presented a total of 42 times. To avoid periodicity, random spacing (40 – 60 ms) was introduced between chirp offsets and onsets. Raised-cosine ramps with durations equal to 10% of chirp duration were applied to each chirp. The sound level of each chirp was set to 65 dB SPL −10 × log_10_(*T*/*T*_*ref*_), where *T* is the duration of the chirp, and *T*_*ref*_ = 2.5 ms (the duration of the ±6.24 kHz/ms chirp). This scaling ensured that energy was normalized among chirps of different durations. To construct the RVF, response rate was calculated by summing spikes over a 15-ms window centered at the peak of the neural response.

For click MTFs, click trains were generated with methods adapted from Godfrey et al. (1975). Rarefaction clicks, 0.1 ms in duration, were generated with rates from 2 – 500 Hz. Click level was approximately 130 dB peSPL, to match the stimuli described in Godfrey et al. (1975). Click MTFs were generated in the same manner as noise MTFs, with rate expressed as a function of click rate. To illustrate entrainment, a special rate calculation was performed to generate click MTFs. Instead of integrating the rate function over the response duration, the number of threshold crossings in the rate function was counted. To ensure each response was only counted once, a refractory period of 1 ms was included. For this calculation only, a threshold of 110 spikes/s was manually selected based on examination of the click-evoked rate functions (this threshold is distinct from the threshold *θ* applied to octopus cell output, described below).

### 2.2 Model Architecture

#### 2.2.1 Model Inputs

Krips and Furst (2009a,b) show that the output of a coincidence detector (CD) is a non-homogeneous Poisson process (NHPP) if it receives independent inputs that are NHPPs. This property of Krips and Furst’s model CDs has the primary advantage of satisfying requirements for the use of statistical decision theory to estimate psychophysical thresholds from model responses, namely that the statistics of discharge patterns are well-described and change as a function of the stimulus parameter of interest (Siebert, 1965; Heinz, 2001a,b). Additionally, Krips and Furst’s method allows for the design of multi-stage model architectures that extend into the central nervous system, with NHPP statistics preserved at each stage.

The CD unit described by Krips and Furst (2009a,b) receives any number of independent inputs, either excitatory or inhibitory, each described by an instantaneous rate function, λ(t). CD units can be defined by two basic interactions of inputs: excitatory-inhibitory (EI) and excitatory-excitatory (EE). A fundamental parameter of both EI and EE interactions is a temporal integration window, Δ. In an EI neuron, Δ describes the time window over which inhibition can suppress the response of the model neuron. In an EE neuron, Δ describes the time window within which excitation from multiple inputs facilitates the model response. To retain NHPP statistics in the output, Δ must be less than the refractory periods of the inputs.

**Fig. 1:**
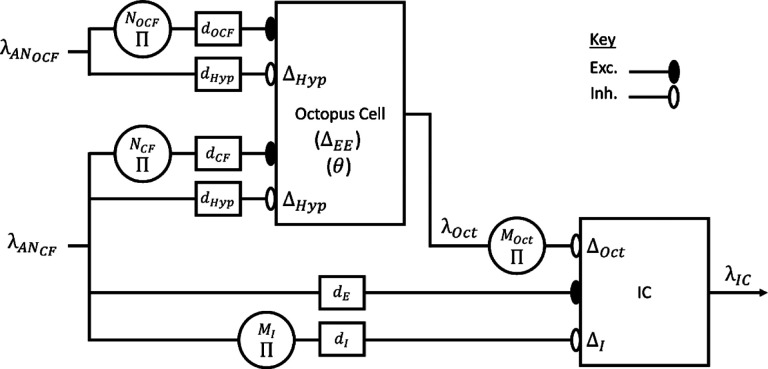
Block Diagram of the model, showing excitatory and inhibitory inputs to both stages. AN input labels indicate whether they are CF or off-CF (OCF) with respect to the CF of the IC neuron. Rate functions are indicated by λ. AN fibers provide CF and off-CF (OCF) excitatory inputs to the octopus cell ($\lambda_{AN_{CF}}$ and $\lambda_{AN_{OCF}}$), delayed by *d*_*CF*_ and *d*_*OCF*_, respectively. The numbers of excitatory inputs are *N*_*CF*_ and *N*_*OCF*_. The excitatory inputs have an integration window Δ_*EE*_. The AN fibers also provide an inhibitory input representing hyperpolarization of the cell due to opening of potassium channels, with delay *d*_*Hyp*_ and integration window Δ_*Hyp*_. Finally, output of the octopus cell below a threshold *θ* was set equal to zero. The output of the octopus cell, λ_*Oct*_, provides *M*_*Oct*_ inhibitory inputs to the IC stage, with integration window Δ_*Oct*_. The IC stage also receives *M*_*I*_ on-CF inhibitory inputs ($\lambda_{AN_{CF}}$) with delay (*d*_*I*_) and integration window Δ_*I*_. Finally, the IC stage receives one CF excitatory input from $\lambda_{AN_{CF}}$, with delay (*d*_*E*_).

The full model introduced here consisted of two distinct Krips-and-Furst CD models, an octopus cell and an IC cell (Fig. 1). Inputs to the octopus-cell stage were provided by a version of the Zilany et al. (2014) AN model that was modified to include gain control via the medial olivocochlear (MOC) efferent (Farhadi et al., 2023) and an improved approximation to the power-law synapse model (Guest and Carney, 2023). The efferent feedback in the AN model affected responses to sounds with modulated envelopes, including the aperiodic random chirp stimulus used here to characterize model neurons’ chirp-velocity sensitivity. Additionally, inclusion of the MOC efferent pathways imparted more physiologically accurate responses to amplitude-modulated noise over a wide dynamic range. The AN model always simulated high-spontaneous-rate fibers, which are the majority of AN fibers (Liberman, 1978). For simplicity, the excitatory input to the IC stage was also provided by a delayed AN response, representing direct inputs from the CN or those relayed through other brainstem nuclei.

The frequencies of the AN fiber inputs were defined as CF and off-CF (OCF), where CF was the desired characteristic frequency of the model IC cell. Note that whether the OCF frequency was above or below CF determined the direction of chirp selectivity. The following sections describe how the two model stages were portrayed using the Krips and Furst (2009a,b) framework, with details provided for how parameter selection related to physiology.

#### 2.2.2 Octopus-Cell Stage

The first stage of the model represented one aspect of octopus-cell responses, velocity sensitivity, based on the sequence detection theory posited by Lu et al. (2022). Sequence detection relies on the KL channels of octopus cells to provide hyperpolarization following either subthreshold or suprathreshold EPSPs. Excitatory inputs that arrive during KL hyperpolarizations did not produce action potentials. To mimic the time-course of hyperpolarization caused by KL channels, we used delayed inhibitory inputs to the octopus cell to represent KL hyperpolarizations. Note that these inputs do not represent the actual inhibitory inputs (from unknown sources) that have been described on octopus cell dendrites (Kreeger et al., 2024).

To implement sequence detection in its simplest configuration, two excitatory AN inputs were used, one subthreshold and one suprathreshold. The CF of the suprathreshold input matched the CF of the model IC cell. The frequency tuning of the off-CF subthreshold input (i.e., whether it was below or above CF) determined the direction of the chirp-velocity selectivity, as described below. The inputs had rate functions $\lambda_{AN_{CF}}$ and $\lambda_{AN_{OCF}}$, respectively. Additionally, the two inhibitory inputs representing KL hyperpolarization were delayed copies of the excitatory AN inputs. Note that since hyperpolarization always occurs after excitation, these inputs are not independent from one another (this issue will be further discussed below).

**Fig. 2:**
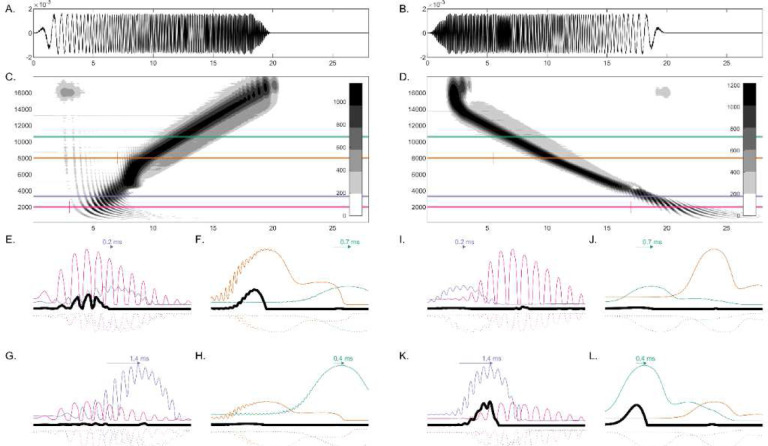
Illustration of sequence-detection mechanism using example chirp responses. **A)** Upward chirp waveform (1.59 kHz/ms, 50 Hz to 16 kHz) **B)** Downward chirp waveform (−1.59 kHz/ms, 16 kHz to 50 Hz) **C)** Neurogram of AN model responses to example upward chirp. Gray shading (color bar) indicates rate function magnitude (lambda). Solid horizontal lines cut through the responses of individual fibers of different CFs (pink = 2 kHz, purple = 3.3 kHz, orange = 8 kHz, green = 10.6 kHz), corresponding to AN inputs to the example octopus-cell responses in E-L. Pink marker at 3 ms and orange marker at 7 ms mark the beginning of the plotted example responses in E-H. Note that the sound level used in this figure was 35 dB SPL (65 dB SPL used elsewhere) to simplify the shape of the neurogram by minimizing spread-of-excitation effects. **D)** Neurogram of AN model responses to example downward chirp. Pink marker at 17 ms and orange marker at 5.5 ms mark the beginning of the plotted example responses in I-L. **E-H)** Responses to an upward chirp of several example octopus cells (black), receiving suprathreshold (thick trace) and subthreshold (thin trace) inputs. Hyperpolarization traces are plotted as dotted lines. Labeled arrows depict delays applied to inputs with matching colors. **I-L)** Responses to a downward chirp of several example octopus cells, in the same format as E-H. Examples E and I receive a 2-kHz suprathreshold input (pink) and 3.3-kHz subthreshold input (purple), resulting in an octopus-cell model with upward chirp selectivity. Examples F and J receive an 8-kHz suprathreshold input (orange) and 10.6-kHz subthreshold input (green), resulting in upward chirp selectivity. Examples G and K receive a 3.3-kHz suprathreshold input (purple) and 2-kHz subthreshold input (pink), resulting in downward chirp selectivity. Examples H and L receive a 10.6-kHz suprathreshold input (green) and 8-kHz subthreshold input (orange), resulting in downward chirp selectivity.

Direction-selectivity of the octopus cell was determined as follows: a chirp eliciting the suprathreshold CF input *before* the subthreshold OCF input resulted in an action potential, because the suprathreshold excitation arrived before KL hyperpolarization could suppress the response. In contrast, a chirp of the opposite direction, eliciting the subthreshold OCF input *before* the suprathreshold CF input, resulted in suppression of the suprathreshold input by the KL hyperpolarization that followed the earlier subthreshold input, resulting in no response. In general, the cell was most responsive to stimuli that excited the suprathreshold (CF) input first. Thus, if the OCF input was tuned higher than IC CF, the octopus cell was selective for upward chirps. Alternatively, if the OCF input was tuned lower than IC CF, the octopus cell was selective for downward chirps (Lu et al., 2022). Fig. 2 illustrates the sequence detection mechanism for two pairs of inputs with different CF ranges.

To better temporally align AN responses with different CFs, which differ in latency, a delay parameter was imposed on both excitatory inputs, denoted as *d*_*CF*_ and *d*_*OCF*_. Depending on the combination of input CFs, the delay was either applied only to the CF input (*d*_*CF*_ > 0 and *d*_*OCF*_ = 0) or only to the OCF input (*d*_*OCF*_ > 0 and *d*_*CF*_ = 0). A delay value that ensured the desired chirp-direction sensitivity across all RVF velocities was determined through parameter optimization (described in detail below). The function of the delays was to ensure two things: one, in response to chirps of the selected-for direction, suprathreshold CF input arrived sufficiently *before* the subthreshold OCF input to avoid suppression by OCF hyperpolarization, and two, in response to chirps of the opposite direction, suprathreshold CF input arrived sufficiently *after* the subthreshold OCF input for maximal suppressed by OCF hyperpolarization. In Fig. 2E–L, arrows above the traces indicate delays that maximized desired direction-selectivity.

The sequence-detection mechanism was implemented using the framework in Krips & Furst (2009a,b), as follows: Consider only the excitatory inputs of the octopus cell ($\lambda_{AN_{CF}}$ and $\lambda_{AN_{OCF}}$). Let *N* = *N*_*CF*_ + *N*_*OC*_, be the number of excitatory AN inputs to the octopus cell, where *N*_*CF*_ represents the number of identical copies of $\lambda_{AN_{CF}}$, and *N*_*OCF*_ represents the number of identical copies of $\lambda_{AN_{OCF}}$. The cell responded when at least *L* inputs are active during an interval Δ. For the purposes of this sequence-detector model, *N*_*CF*_ = *L* and *N*_*OCF*_ = 1, and Δ is Δ_*EE*_ (Fig. 1); therefore, the model cell responded only if activity occurred on at least *N* − 1 inputs during an interval Δ_*EE*_. Furthermore, *N*_*CF*_ > *N*_*OCF*_ because the suprathreshold input, $\lambda_{AN_{CF}}$, had to trigger an action potential in isolation, and $\lambda_{Oct_{OCF}}$ had to be subthreshold in isolation.

This octopus cell can be initially thought of as a multiple-input EE cell that responds either when receiving exactly *N* active inputs or when receiving exactly *N* − 1 active inputs. Let us consider the first case, when the cell receives *L* = *N* active inputs. The set of all inputs is {λ_1_,…, λ_*N*_}. The instantaneous rate is described by (Eqn. 4.21 in Krips and Furst, 2009b):

$$\lambda_{EE_{L}(t)}=\sum_{l=1}^{L}\;\lambda_{l}(t)\prod_{j=1,j\neq l}^{L}\;\int_{t-\Delta }^{t}\;\lambda_{j}\left(t^{\prime }\right)dt^{\prime }.$$

The cell responds at time *t* only when activity is observed on all *L* = *N* inputs within the time interval (*t* − Δ, *t*). Note that here, Δ = Δ_*EE*_.

Now consider the second case, when the cell receives exactly *N* − 1 active inputs. Let *l* be the exact number of active inputs. The *i*th set of active inputs is denoted as $\Psi_{l_{i}}=\left\{{\lambda_{1}}^{\left(i\right)},\ldots ,{\lambda_{l}}^{\left(i\right)}\right\}$. The complementary set of *N* − *l* inactive inputs is denoted as $\Omega_{l_{i}}=\left\{{\lambda_{l+1}}^{\left(i\right)},\ldots ,{\lambda_{N}}^{\left(i\right)}\right\}$. If *L* ≤ *l* ≤ *N*, there are Nl (*N*-choose-*l*) sets of active inputs possible. Therefore, the instantaneous rate of a cell that responds when exactly *l* inputs are active, and *N* − *l* inputs are not active, is described by (Eqn. 4.23 in Krips and Furst, 2009b):

λEE=lN(Ψ)=∑i=1Nl λEElΨliλIΩli,

where $\lambda_{EE_{l}}\left(\Psi_{l_{i}}\right)$ is the instantaneous rate of the EE cell receiving the set of active inputs $\Psi_{l_{i}}$, given by Eqn. 4.21 in Krips and Furst (2009b). Meanwhile, $\lambda_{I}\left(\Omega_{l_{i}}\right)$ is the instantaneous rate of the set of inactive inputs $\Omega_{l_{i}}$, described by (Eqn. 4.24 in Krips and Furst, 2009b):

$$\lambda_{I}\left(\Omega_{l_{i}}\right)=\prod_{j=l+1}^{N}\left(1-\int_{t-\Delta }^{t}\lambda_{j}(i)\left(t^{\prime }\right)dt^{\prime }\right).$$

The octopus cell responds if *l* = *N* or *l* = *N* − 1. The instantaneous rate of each of these two cases is found by substituting *l* into Eqn. 4.23 in Krips and Furst (2009b). Summing these two functions gives the final instantaneous rate of the multiple-input EE cell, $\lambda_{EE_{L}^{N}}(\Psi )$.

Next, the effect of the KL hyperpolarization inputs can be considered. These inhibitory inputs are copies of the excitatory inputs, $\lambda_{AN_{CF}}$ and $\lambda_{AN_{OCF}}$, delayed by *d*_*Hyp*_ seconds. Letting Ψ_*AN*_ be the set of all AN inputs (where again, *N* = *N*_*CF*_ + *N*_*OCF*_), the full equation for the instantaneous rate of the octopus cell stage is described by (Eqn. 1):

(Eqn 1) $$\lambda_{Oct}(t)=\lambda_{EE_{L}^{N}\left(\Psi_{AN}\right)}\cdot \left(1-\int_{t-\Delta_{Hyp}}^{t}\lambda_{AN_{CF}}\left(t^{\prime }\right)dt^{\prime }\right)\;\cdot \left(1-\int_{t-\Delta_{Hyp}}^{t}\lambda_{AN_{OCF}}\left(t^{\prime }\right)dt^{\prime }\right).$$

Finally, to ensure that the octopus cell has an “ideal onset” quality (Godfrey et al., 1975; Rhode and Smith, 1986; Oertel et al., 2000), a threshold *θ* was applied to λ_*Oct*_, that is, samples of λ_*Oct*_(*t*) below *θ* were set to zero. The value of *θ* was chosen by observing rate functions of the octopus cell stage in response to click trains. The value *θ* = 50 spk/s eliminated activity between click cycles.

#### 2.2.3 IC Stage

The second stage of the model represented a neuron in the IC that received excitatory input from the brainstem and inhibition from the octopus-cell, which gave it chirp-direction sensitivity. The IC model neuron also received a delayed inhibitory input with the same CF as the excitatory input, as in the same-frequency inhibition and excitation (SFIE) model for AM tuning (Nelson and Carney, 2004).

The AM tuning of a neuron is characterized by a modulation transfer function (MTF), the average response rate versus modulation frequency. The SFIE model produces neurons with BE MTFs. For the IC stage here, the brainstem was not explicitly modeled, for simplicity, and the excitatory brainstem input was represented by a version of $\lambda_{AN_{CF}}$ that was delayed by *d*_*E*_. The corresponding inhibition was also represented by a copy of $\lambda_{AN_{CF}}$ that was delayed by *d*_*I*_. This inhibition had the associated parameters Δ_*I*_, the integration window, and *M*_*I*_, describing the number of times the inhibitory input was duplicated. To ensure the octopus-cell inhibition arrived before the excitatory input, the value of *d*_*E*_ was greater than 0. Additionally, to ensure the same-frequency inhibition arrived after the excitatory input, the inhibitory delay, *d*_*I*_, was greater than the excitatory delay, *d*_*E*_.

The inhibition from the octopus-cell stage, λ_*Oct*_, had its own set of parameters: *d*_*Oct*_, Δ_*Oct*_, and *M*_*Oct*_, for the delay, integration window, and number of inhibitory inputs, respectively. The instantaneous rate at the IC stage output was defined as

(Eqn 2) $$\lambda_{IC}\left(t\right)=\lambda_{AN_{CF}}\left(t\right)\cdot M_{I}\left(1-\int_{t-\Delta_{I}}^{t}\lambda_{AN_{CF}}\left(t^{\prime }\right)dt^{\prime }\right)\cdot M_{Oct}\left(1-\int_{t-\Delta_{Oct}}^{t}\lambda_{Oct}\left(t^{\prime }\right)dt^{\prime }\right).$$

Additionally, the final output λ_*IC*_ was half-wave rectified to prevent negative rates. As illustrated below, the model IC cell was sensitive to chirp direction and velocity and had BE AM tuning.

#### 2.2.4 Parameter Selection

The chirp-velocity sensitivity of the model IC cell was dependent upon the sensitivity of the octopus-cell inhibitory input. Therefore, the selection of octopus-cell parameters was important for generating model IC cells with physiologically appropriate chirp responses. Sensitivity towards chirp velocity and direction can be characterized by RVFs. While octopus-cells have heterogeneous chirp sensitivity (Lu et al., 2022), and thus would have a variety of RVF shapes, for the purpose of this study it was useful to consider two basic types, one selective for upward chirps and one for downward chirps.

Octopus-cell parameters were determined using the MATLAB parameter-optimization tool *fmincon* (2022a, MathWorks). This tool is designed to determine the parameters that minimize the output of a loss function. Here, the loss function was 1 − *corr*(*RVF*_*mod*_, *RVF*_*tem*_), where *RVF*_*mod*_ was the RVF of the model octopus cell, *RVF*_*tem*_ was the template RVF, and *corr* was the linear correlation operation (note that this loss function was identical to maximizing the correlation between model and template RVFs). Two template RVFs were used: the upward-selective template had rates of one for positive velocities and rates of zero for negative velocities; the downward-selective template had rates of one for negative velocities and zero for positive velocities. These two simple RVF shapes were chosen to impart the most basic direction selectivity upon the octopus-cell RVFs.

Octopus-cell parameters yielding upward-selective and downward-selective RVFs were found for CFs of 1, 4, and 8 kHz (representing low, medium, and high IC CFs). To ease optimization, the octopus-cell parameter space was simplified to two free parameters: OCF, the frequency of the off-CF input, and a single delay that was applied to the higher-CF of the two AN inputs, either *d*_*CF*_ or *d*_*OCF*_. Initial parameter values were randomly selected within each parameter’s lower and upper bounds (Table 1). Note that the bounds for OCF depended on the desired direction-selectivity of the octopus cell. From here, *fmincon* optimized the free parameters that minimized the objective function and resulted in an RVF that most resembled the template RVF.

For the remaining octopus-cell parameters, *N*_*CF*_, Δ_*EE*_, Δ_*Hyp*_, and *d*_*Hyp*_, a range of values was explored to optimize the chirp-sensitivity of octopus-cell RVFs. This exploration is summarized in Results (Figs. 7–10**Error! Reference source not found.**), and the default values for each parameter are given in Table 1.

Finally, IC-stage parameters *d*_*Oct*_, *M*_*Oct*_, *d*_*I*_, and *M*_*I*_, were manually selected to match the desired response properties, i.e., an IC cell receiving upward-selective octopuscell inhibition had parameters to maximize downward-selectivity in its RVF. Parameters of all IC cells were selected to yield BE MTFs. IC velocity-sensitivity was primarily affected by octopus-cell inhibition parameters (*d*_*Oct*_, *M*_*Oct*_), and periodicity tuning was primarily affected by SFIE inhibition parameters (*d*_*I*_, *M*_*I*_). The impact of varying these IC-stage parameters is summarized in Figs. 11–14. For simplicity, Δ_*Oct*_ and Δ_*I*_ were both set equal to 1 ms.

## 3 Results

### 3.1 Octopus-Cell Stage Responses

**Fig. 3:**
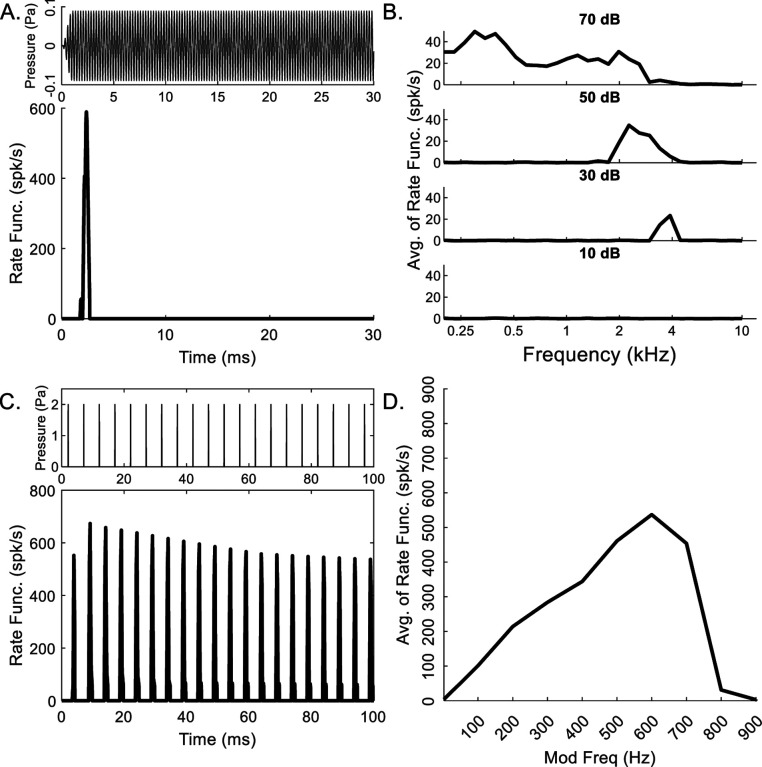
Responses of an example model octopus cell to tone and click stimuli. A) Rate function (λ_*Oct*_) in response to a pure tone at CF, B) Pure-tone response map, illustrating CF at 4 kHz, C) Rate function in response to a click train (200 Hz, peak level 130.6 dB peSPL), and D) Click MTF for modulation frequencies from 2–900 Hz. For this panel only, to illustrate entrainment, threshold-crossings of the rate-function (C) were counted to approximate action potentials (threshold = 110 spk/s).

Tones and click stimuli were used to confirm that response properties of the octopus-cell stage were consistent with physiological recordings (Godfrey et al., 1975; Rhode and Smith, 1986). The responses of an upward-sensitive octopus cell (λ_*Oct*_) with CF = 4 kHz illustrate a rate function with a strong onset response to a pure tone at CF, followed by rates near zero (Fig. 3A).

The pure-tone RM (Fig. 3B) reflects the CF of 4 kHz, with a broader frequency-response at 50 and 70 dB SPL. In response to a click train, a peak in the rate function was observed for every click; the amplitude of the rate function increased at the beginning of the response but leveled off with time (Fig. 3C). With increasing click rate, the response rate entrained until 600 Hz, and stopped responding at 900 Hz (Fig. 3D).

### 3.2 Example Neurons

Example neurons with both upward and downward chirp-direction sensitivity were produced with low, medium, and high CFs. Parameter values for example neurons are provided in Table 2. Note that these parameters were manually selected to result in example model neurons with substantial chirp sensitivity and AM tuning that was representative of IC recordings (Mitchell et al., 2023). Optimal fitting of this large set of parameters to actual neural responses may be possible but is beyond the scope of this study.

**Fig. 4:**
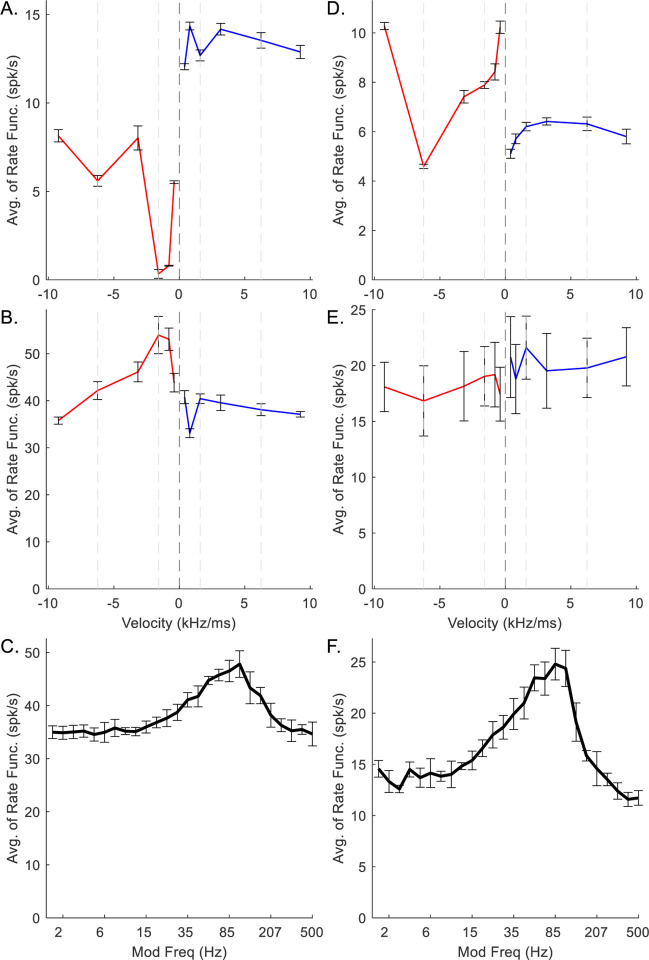
Example low-CF (1 kHz) octopus-cell (A, D) and IC (B,C,E,F) model responses. A-C correspond to upward-sensitive octopus and downward-sensitive IC models, D-E correspond to downward-sensitive octopus and upward-sensitive IC models. In RVFs, blue indicates response to upward velocities, red indicates response to downward velocities. Error bars indicate standard deviation of 5 model repetitions.

For model IC neurons with low CF (CF = 1 kHz) (Fig. 4), direction sensitivity was less prominent than for higher-CF neurons. Direction sensitivity is observed in the RVF plots by comparing the rates in response to positive and negative chirp directions at each chirp speed. In the plots below, vertical dashed lines at ±1.59 and ±6.24 kHz/ms have been included for ease of comparison. The downward-sensitive IC neuron received upward-sensitive octopus-cell inhibition (Fig. 4A). The model octopus cell was upward-sensitive across all velocities in the RVF, whereas the model IC cell was downward-sensitive for chirps below ±3.16 kHz/ms, and was not direction sensitive at higher speeds (Fig. 4B). The MTF of the IC stage was BE (Fig. 4C).

The upward-sensitive model IC neuron received downward-sensitive octopus-cell inhibition (Fig. 4D). The RVF of the octopus-cell inhibition was uniformly downward-sensitive, with the exception of ±6.24 kHz/ms; the RVF of the IC stage was weakly upward-sensitive (Fig. 4E). The MTF of the IC stage was BE (Fig. 4F). Notably, for both low-CF examples, *N*_*CF*_ was set to 4, resulting in stronger chirp-sensitivity (*N*_*CF*_ was set to 3 for model neurons with higher CFs, below). The error bars indicate the standard deviation of 5 model trials. Both octopus-cell RVFs have small error bars (Fig. 4A, D), whereas the IC RVFs have relatively large error bars, indicating greater variability between runs of the model (Fig. 4B, E). Specifically, the upward-sensitive IC RVF (Fig. 4E) has velocity pairs with overlapping error bars. This high variability reflected the relatively greater difficulty in finding suitable parameters to portray direction sensitivity for low-CF neurons compared to higher-CF neurons.

**Fig. 5:**
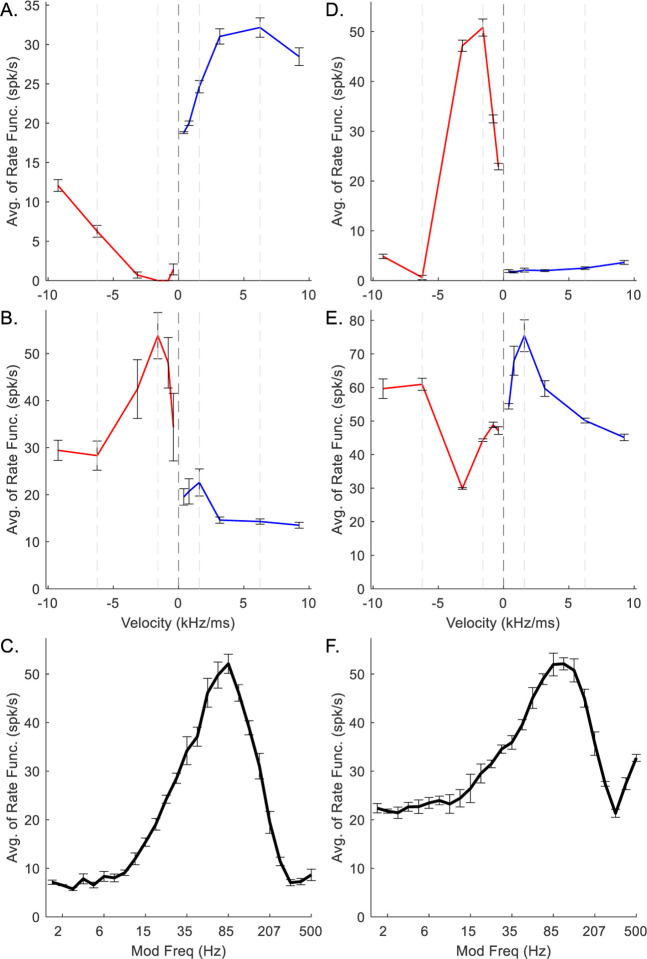
Example medium-CF (4 kHz) octopus-cell (A, D) and IC (B,C,E,F) model responses. A-C correspond to upward-sensitive octopus and downward-sensitive IC models, D-E correspond to downward-sensitive octopus and upward-sensitive IC models. In RVFs, blue indicates response to upward velocities, red indicates response to downward velocities. Error bars indicate standard deviation of 5 model repetitions.

Chirp-sensitive medium-CF (CF = 4 kHz) model neurons (Fig. 5) had MTFs with more prominent peaks and RVFs with larger rate-differences between directions than the low-CF neurons (Fig. 4). The RVF of the upward-sensitive medium-CF octopus cell had large rate-differences for all velocity pairs (Fig. 5A). The rate-differences in the downward-sensitive IC RVF are also large (Fig. 5B). The IC MTF (Fig. 5C) was BE, with a well-defined peak at about 100 Hz. In contrast to the upward-sensitive octopus cell (Fig. 5A), the downward-sensitive medium-CF octopus-cell (Fig. 5D) was upward-sensitive at low chirp speeds (<3.16 kHz/ms), but not at high speeds (>6.24 kHz/ms). Similarly, the IC RVF (Fig. 5E) is downward-sensitive for low chirp speeds, and upward-sensitive at high speeds. The IC MTF is BE, with a BMF of about 100 Hz (Fig. 5F). For both medium-CF model cells, direction-sensitivity was strongest for velocities below ±3.16 kHz/ms.

**Fig. 6:**
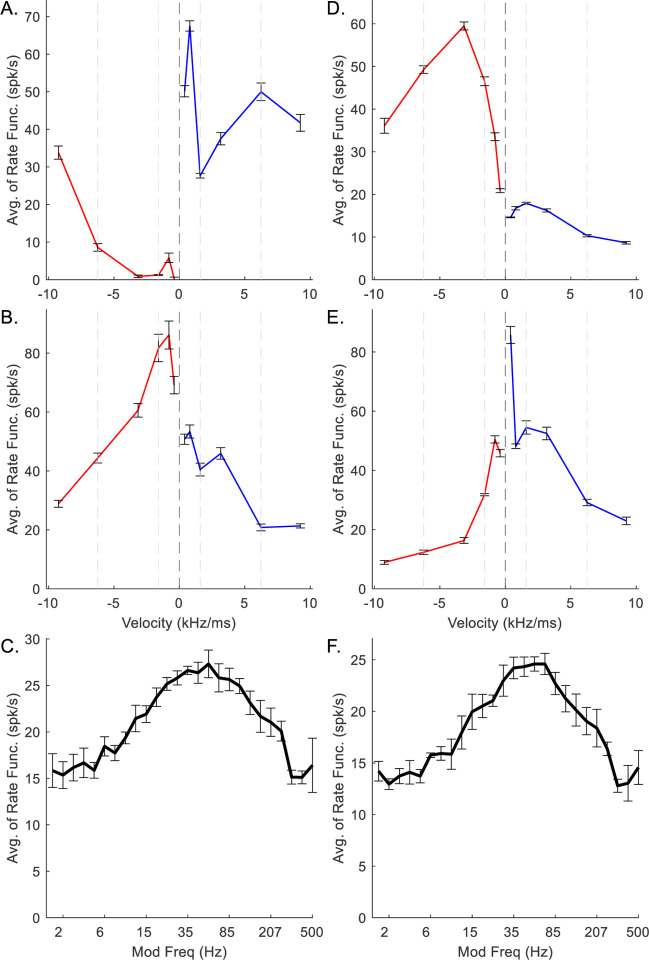
Example high-CF (8 kHz) octopus-cell (A, D) and IC (B,C,E,F) model responses. A-C correspond to upward-sensitive octopus and downward-sensitive IC models, D-E correspond to downward-sensitive octopus and upward-sensitive IC models. In RVFs, blue indicates response to upward velocities, red indicates response to downward velocities. Error bars indicate standard deviation of 5 model repetitions.

Chirp-sensitive high-CF (CF = 8 kHz) IC neurons (Fig. 6) had slightly smaller rate-differences between directions than CF = 4 kHz (Fig. 5). The RVF of the octopus-cell (Fig. 6A) that inhibited the downward-sensitive IC neuron was upward-sensitive at all velocities, with response rate peaking at +0.80 kHz/ms. The corresponding IC RVF (Fig. 6B) is downward-sensitive at all velocities, but with smaller rate differences than the medium-CF example (Fig. 5B). The IC MTF is BE (Fig. 6D), with BMF around 40 Hz. Finally, the downward-sensitive octopus cell has strong direction sensitivity for all velocities (Fig. 6D). The IC RVF (Fig. 6E) is upward-sensitive, except for ±0.80 kHz/ms, for which it is slightly downward-sensitive. Also notable is the large rate-difference for ±0.40 kHz/ms, despite the comparatively smaller rate difference in the octopus cell RVF (Fig. 6E). The IC MTF is BE (Fig. 6F), with a BMF of approximately 40 Hz.

### 3.3 Effect of Varying Parameters

Parameters for example cells were selected with the goal of maximizing chirp-direction sensitivity and BE MTF tuning. The following section illustrates the contribution of each parameter to the model responses to chirps and AM noise.

**Fig. 7:**
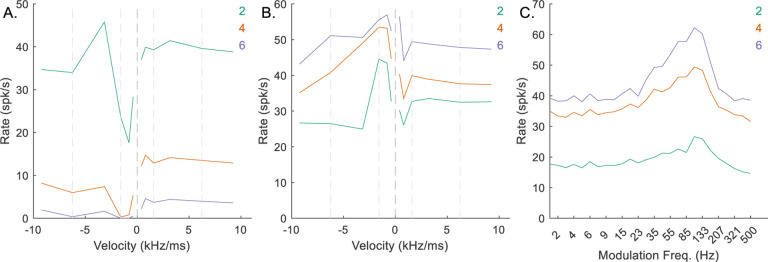
Impact of varying *N*_*CF*_ on responses of an example low-CF (1 kHz) model cell with downward-sensitive IC output (Green – *N*_*CF*_ = 2, orange – *N*_*CF*_ = 4, purple – *N*_*CF*_ = 6). Responses for *N*_*CF*_ = 4 are also shown in Figs. 4A–C. A) octopus cell RVF; B) IC RVF; C) IC MTF.

*N*_*CF*_ represented the number of identical on-CF excitatory inputs arriving to the octopus cell, and *N*_*CF*_ = *L*, the number of active inputs required for the octopus cell to respond. For the low-CF, upward-sensitive, octopus-cell example, decreasing *N*_*CF*_ from 4 to 2 resulted in increased rates in response to all stimuli (Fig. 7A), ultimately making the model IC neuron less downward-sensitive (Fig. 7B), and reducing the amplitude of the peak in the BE MTF (Fig. 7C). Conversely, increasing *N*_*CF*_ from 4 to 6 reduced the octopus-cell response rates (Fig. 7A), resulting in less octopus-cell inhibition to the IC cell, and again a less downward-chirp sensitive RVF (Fig. 7B). It was apparent that there was an optimal value for *N*_*CF*_ that maximized the desired chirp-direction sensitivity. For mid-and-high-CF neurons, this value was 3, whereas for low-CF neurons, *N*_*CF*_ = 4 resulted in the strongest direction sensitivity.

**Fig. 8:**
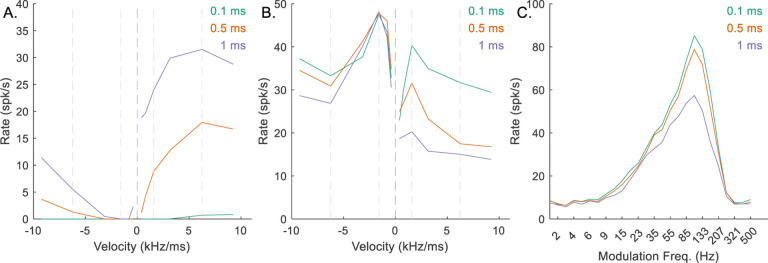
Impact of varying Δ_*EE*_ on responses of an example mid-CF (4 kHz) model cell with downward-sensitive IC output (Green – Δ_*EE*_ = 0.1 ms, orange – Δ_*EE*_ = 0.5 ms, purple – Δ_*EE*_ = 1 ms). Responses for Δ_*EE*_ = 1 ms are also shown in Figs. 5A–C. A) octopus cell RVF; B) IC RVF; C) IC MTF.

**Fig. 9:**
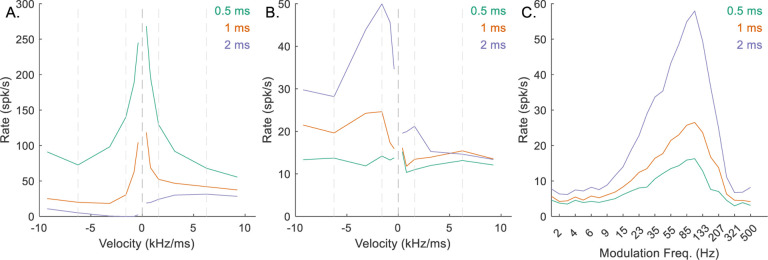
Impact of varying Δ_*Hyp*_ on responses of an example mid-CF (4 kHz) model cell with downward-sensitive IC output (Green – Δ_*Hyp*_ = 0.5 ms, orange – Δ_*Hyp*_ = 1 ms, purple – Δ_*Hyp*_ = 2 ms). Responses for Δ_*Hyp*_ = 2 ms are also shown in Figs. 5A–C. A) octopus cell RVF; B) IC RVF; C) IC MTF.

**Fig. 10:**
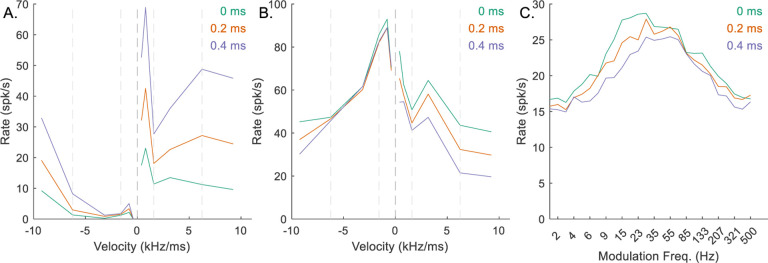
Impact of varying *d*_*Hyp*_ on responses of an example high-CF (8 kHz) model cell with downward-sensitive IC output (Green – *d*_*Hyp*_ = 0 ms, orange – *d*_*Hyp*_ = 0.2 ms, purple – *d*_*Hyp*_ = 0.4 ms). Responses for *d*_*Hyp*_ = 0.4 ms are also shown in Figs. 6A–C. A) octopus cell RVF; B) IC RVF; C) IC MTF.

In general, Δ described the integration windows of CDs. Per Krips and Furst (2009b), this value should be smaller than the refractory period of the neuron. However, the integration window for the EE inputs to the octopus cell (Δ_*EE*_) had to be 1 ms or greater to capture the desired chirp-direction sensitivity in either the octopus RVF (Fig. 8A, purple line) or the IC RVF (Fig. 8B, purple line); smaller integration windows resulted in less sensitive neurons (Fig. 8B, orange and green lines). Additionally, the integration window of the hyperpolarization inhibition (Δ_*Hyp*_) had to be relatively long to adequately suppress the excitatory signals (Fig. 9). Values of Δ_*Hyp*_ less than approximately 2 ms resulted in octopus-cell RVFs that were not direction sensitive (Fig. 9A, green and orange), and ultimately non-sensitive IC RVFs (Fig. 9B, green and orange). Implications of these integration window values will be discussed below. Finally, the value of the hyperpolarization delay, *d*_*Hyp*_, affected octopus-cell chirp sensitivity (Fig. 10A) and sensitivity of the IC RVF (Fig. 10B), with short delay associated with reduced direction sensitivity.

**Fig. 11:**
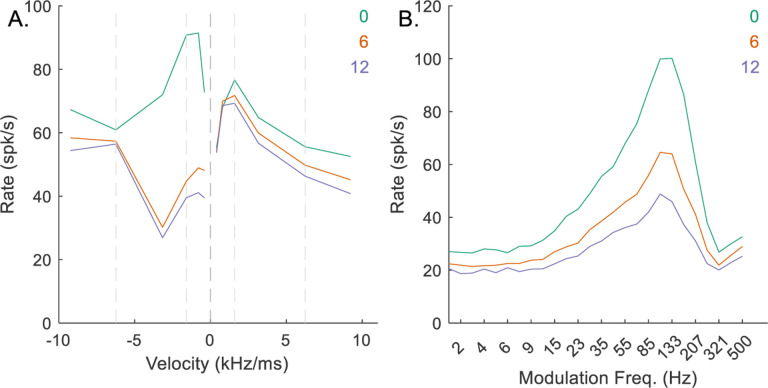
Impact of varying *M*_*Oct*_ on responses of an example mid-CF (4 kHz) model cell with upward-sensitive IC output (Green – *M*_*Oct*_ = 0, orange – *M*_*Oct*_ = 6, purple – *M*_*Oct*_ = 12). Responses for *M*_*Oct*_ = 6 are also shown in Figs. 5D–F. A) IC RVF; B) IC MTF.

At the IC stage, chirp-sensitivity was primarily controlled by the parameters *M*_*Oct*_ and *d*_*E*_, the number of inhibitory octopus-cell inputs and the delay of the excitatory input relative to the octopus-cell inputs. For the mid-CF, upward-sensitive, example IC neuron, when *M*_*Oct*_ was set at zero, there was no impact of the octopus cell on the RVF (Fig. 11A), but the MTF had a large rate at the peak (Fig. 11B). As *M*_*Oct*_ increased, the RVF became upward-sensitive (Fig. 11A), but the MTF rate decreased (Fig. 11B), demonstrating that *M*_*Oct*_ selection must balance the desired chirp-direction sensitivity and prominence of the BE MTF.

**Fig. 12:**
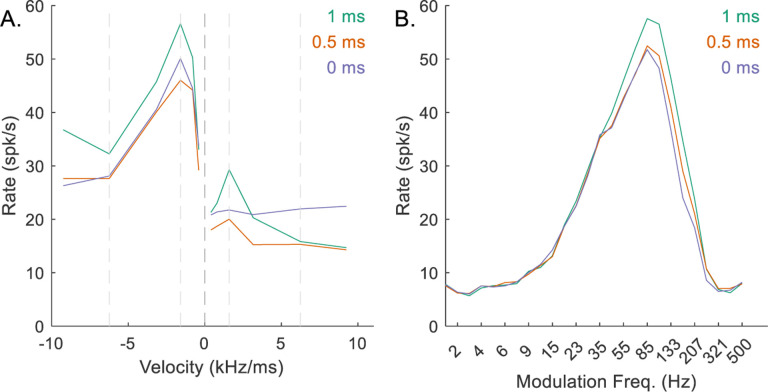
Impact of varying *d*_*E*_ on responses of an example mid-CF (4 kHz) model cell with downward-sensitive IC output (Green – *d*_*E*_ = 1.5 ms, orange – *d*_*E*_ = 1 ms, purple – *d*_*E*_ = 0.5 ms). Responses for *d*_*E*_ = 0.5 ms are also shown in Figs. 5A–C. A) IC RVF; B) IC MTF.

To maximize IC chirp-direction sensitivity, *d*_*E*_ must allow octopus-cell inhibition to arrive sufficiently before excitation. For the example mid-CF, downward-sensitive neuron, *d*_*E*_ = 0.5 ms resulted in the largest downward-chirp sensitivity (Fig. 12A). Using a higher value of *d*_*E*_, such as 1 ms, reduced downward-chirp sensitivity (Fig. 12A).

**Fig. 13:**
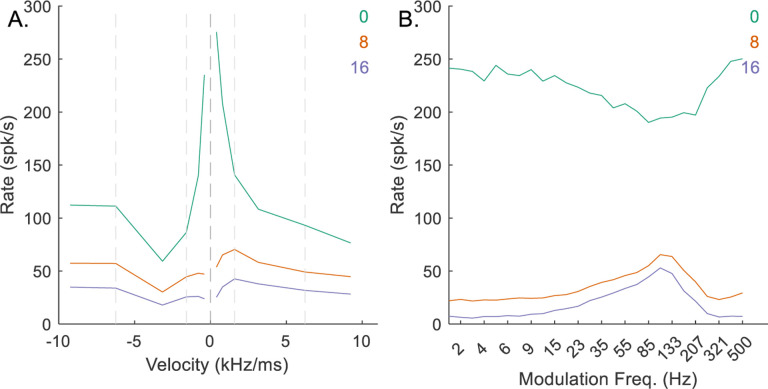
Impact of varying *M*_*I*_ on responses of an example mid-CF (4 kHz) model cell with upward-sensitive IC output (Green – *M*_*I*_ = 0, orange – *M*_*I*_ = 8, purple – *M*_*I*_ = 16). Responses for *M*_*I*_ = 8 are also shown in Figs. 5D–F. A) IC RVF; B) IC MTF.

The AM-tuning of the model IC neuron was controlled by *M*_*I*_ and *d*_*I*_: *M*_*I*_ defined the number of inhibitory CF inputs the neuron received, and *d*_*I*_ defined the delay of the CF inhibition relative to the octopus-cell inputs, where *d*_*I*_ − *d*_*E*_ was the delay between CF excitation and CF inhibition. If *M*_*I*_ were zero, the tuning of the MTF was not affected by same-frequency inhibition (Fig. 13B), instead having a flat or possibly band-suppressed MTF shape. Increasing *M*_*I*_ gave the MTF a BE shape and sharpened the peak (Fig. 13B), and, as expected, reduced the response rates across all stimuli (Fig. 13A).

**Fig. 14:**
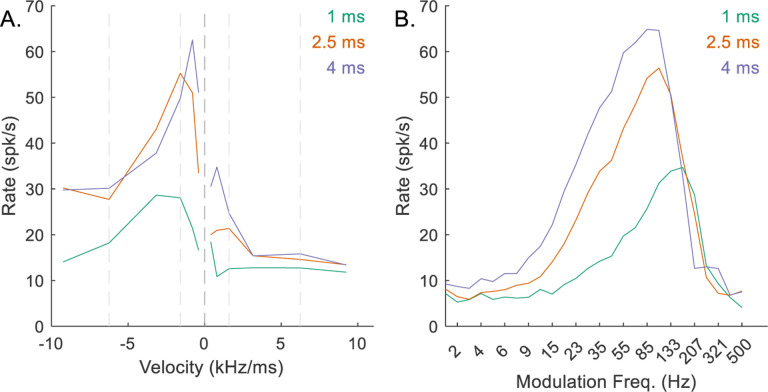
Impact of varying *d*_*I*_ − *d*_*E*_ on responses of an example mid-CF (4 kHz) model cell with downward-sensitive IC output (Green – *d*_*I*_ − *d*_*E*_ = 1 ms, orange – *d*_*I*_ − *d*_*E*_ = 2.5 ms, purple – *d*_*I*_ − *d*_*E*_ = 4 ms). Responses for *d*_*I*_ − *d*_*E*_ = 2.5 ms are also shown in Figs. 5A–C. A) IC RVF; B) IC MTF.

The delay between CF excitation and inhibition, *d*_*I*_ − *d*_*E*_, determined the location of the MTF peak. For the example mid-CF neuron, as this value increased, the BE peak shifted to lower modulation frequencies (Fig. 14B). The RVF was also affected when the delay was small (Fig. 14A), illustrating an interaction between the peak modulation frequency of the MTF and the shape of the RVF.

## 4 Discussion

These results describe a computational model for both chirp-sensitivity and periodicity tuning in the IC. Chirp-sensitivity in the model originated in model PVCN octopus cells, which also had characteristics such as O_I_ tone responses and click entrainment. Depending on parameter values, IC cells with sensitivity towards either chirp direction and with BE-type MTFs could be simulated for low, medium, and high CFs. Chirp-sensitivity and periodicity tuning were largely controlled by separate inhibitory parameters in the IC. Model parameters had systematic effects on IC RVFs and MTFs, allowing responses to be tuned.

Responses to tone and click stimuli confirmed that the octopus-cell stage was consistent with this cell type’s physiological responses (Fig. 3), although the primary purpose of this stage was to provide a chirp-sensitive input to the IC. Octopus cells have an ideal onset (O_I_) response to high-frequency tones, with one well-timed response at tone onset (Godfrey et al., 1975; Rhode and Smith, 1986). The model octopus cell responded to a tone at CF with a peak in the rate function shortly after tone onset, followed by no activity. Frequency-response areas of octopus cells are broad, consistent with their wide-dendritic fields (Osen, 1969; Rhode et al., 1983). O_I_ cells tend to have thresholds greater than 30 dB, with much higher rates at frequencies lower than CF and at high sound levels (Rhode and Smith, 1986; Rhode, 1994), as does the model RM (Fig. 3B). Octopus cells are also characterized by entrainment to modulated stimuli such as click trains, responding with one precisely timed action potential for every cycle for frequencies up to 500–800 Hz (Godfrey et al., 1975; Rhode, 1994; Oertel et al., 2000), similar to model responses (Fig. 3C). Finally, the model click MTF (Fig. 3D) increased monotonically up to 600 Hz, a slightly lower frequency than observed in O_I_ neurons, which entrained up to 700 Hz to clicks of a comparable level (Godfrey et al., 1975).

The results of this modeling study agree with the physiological results of our previous study of the responses of rabbit IC neurons to chirp stimuli (Mitchell et al., 2023). Diverse chirp sensitivity was observed across all CF ranges (Mitchell et al., 2023). Here, we show that it was possible to choose model parameters to produce chirp sensitivity for a similar range of CFs. In physiology, chirp-direction sensitivity is more common towards low-speed chirps (< 2 kHz/ms) than towards high-speed chirps (> 2 kHz/ms), a result also observed in model responses (Fig. 4–6). For many example IC model responses, rate-differences between the fastest chirp-pairs were smaller than for slower chirp-pairs (Fig. 5B), and sometimes displayed bias towards the opposite direction (Fig. 5E). This property of RVFs at high chirp-velocities, also observed in some physiological responses (e.g., Fig. 4D, Mitchell et al., 2023), may occur when chirps of opposing directions begin to resemble each other as representations in the AN become more click-like.

Also, Mitchell et al. (2023) suggested that chirp-direction sensitivity and periodicity tuning in the IC originate from different neural mechanisms that create two distinct feature sensitivities. This conclusion was echoed in the modeling results—chirp-sensitivity in the model was strongest when octopus-cell inhibition arrived about 1 ms before the IC excitatory input (Fig. 12), and BE MTF peaks were highest when the same-frequency inhibition arrived more than 1 ms after IC excitation (Fig. 14). That these inhibitions functioned best when their timings did not coincide suggests that they come from separate inputs.

Krips and Furst (2009a,b) showed that CD responses are NHPPs for an integration window Δ much smaller than the refractory period of their inputs. They suggested a Δ of 200 μs for EI CDs and 20 μs for EE CDs (Krips and Furst, 2009a). The rationale for the limitation of Δ is to prevent multiple spikes from the same input from occurring within the integration window, thereby triggering a response without a coincidence from multiple inputs. However, here, a small Δ_*EE*_ (≤ 100 μs) resulted in the model octopus cell, and subsequently the model IC neuron, having greatly reduced chirp-direction sensitivity (Fig. 8). A short Δ_*Hyp*_, the hyperpolarization integration window, also reduced chirp sensitivity (Fig. 9). Due to the long integration windows used in the model, which appear to be necessary to ensure chirp-direction sensitivity, the model output would not be a NHPP. This is a limitation of the current model, potentially preventing its use in estimating psychophysical detection thresholds for various stimulus parameters. However, the prerequisite for long integration windows would be expected for a model of a chirp-direction sensitive neuron with widely spaced CF inputs. Conceptually, there is a natural trade-off between difference in input CF and length of integration window—when inputs are far apart in CF, they necessarily require a larger Δ. Also, KL hyperpolarizations have relatively long timescales compared to the suggested integration windows (Golding et al., 1995). Long Δ_*Hyp*_ may be unavoidable if treating KL hyperpolarization as an inhibitory input, as was done here. In the future, it might be possible to identify sets of model parameters that allow shorter integration windows but still retain chirp-sensitivity. Furthermore, hyperpolarization inhibition cannot be treated as independent from its corresponding excitatory input as it is entirely conditional upon the excitatory activity. In future work, this limitation could be addressed by combining the “locked” excitatory AN input and inhibitory hyperpolarization into a complex “excitatory-inhibitory” signal more closely representing the full post-synaptic response.

Parameters for the example neurons were selected, using a combination of parameter optimization and manual selection, to maximize direction-sensitivity. The values of these parameters align well with their physiological correlates. For instance, octopus cells provide early onset inhibition to the IC via the VNLL (Covey and Casseday, 1991). Intracellular recordings in VNLL and IC cells showed that IC cells that received VNLL inhibition often displayed an early inhibition before action potentials in response to sounds (Nayagam et al., 2005). In the present study, inhibition arriving from the octopus-cell stage 0.5–1 ms before the excitatory input maximized the chirp sensitivity (Fig. 12). Additionally, if this inhibition arrived too early or too late relative to excitation, chirp sensitivity was diminished. Similarly, the CFs of AN inputs to the octopus cell stage were aligned with experimental and modeling studies, which show that these CFs can come from a wide range of frequencies: Spencer et al. (2012) estimated CFs of AN inputs to octopus cells based on physiological recordings in cats, and determined they can range from 1.5 – 40 kHz (Godfrey et al., 1975; Rhode and Smith, 1986).

In this paper, octopus cells were proposed as a source of chirp-velocity sensitivity for IC cells; however, alternative mechanisms have been proposed for frequency-modulation (FM) sensitivity. For example, Pollak et al. (2011) summarized two mechanisms other than VNLL-inhibition that could explain sensitivity of IC neurons to FM chirps. One of these is the classical explanation for FM sensitivity, based on asymmetry in the timing and frequency of excitation and inhibition (Fuzessery and Hall, 1996; Gordon and O’Neill, 1998; Andoni et al., 2007). This asymmetry is revealed by spectrotemporal receptive fields (STRFs), a technique using spike-triggered averaging to generate a kernel used to identify excitatory and inhibitory regions. STRFs have been shown to predict the sensitivity of chirp-direction sensitive neurons in bat IC (Andoni et al., 2007). Another hypothesis for chirp sensitivity in the IC proposes that cells with high input resistances and long time constants could be sensitive to asymmetry in input magnitudes, rather than input timing (Gittelman et al., 2009). The modular nature of Krips and Furst’s modeling strategy may facilitate exploration of these additional chirp-sensitivity mechanisms.

In physiological recordings, chirp-sensitive neurons with band-suppressed (BS) MTFs, characterized by lower rates in response to modulated stimuli compared to unmodulated stimuli, are at least as common as BE ones (Mitchell et al., 2023). It may be possible to model chirp-sensitive neurons with BS MTFs by using a similar strategy to Carney et al. (2015), which used an inhibitory input from a BE model cell. Implementing an inhibitory interneuron would require careful calibration of additional timing parameters but may be a useful advancement towards understanding the responses of all chirp-sensitive IC neurons.

The results shown here involved manual selection of IC parameters, with the goal of maximizing direction-sensitivity in the RVF; other response characteristics, such as the salience of MTF tuning, could be increased at the expense of direction-sensitivity. In general, the model parameter space is open-ended, with a potential to simulate neurons with differing response features. A strategy of parameter fitting could eventually be employed to simulate actual IC neuron recordings. Additionally, keeping in mind the sensitivity of these neurons to interaural differences, one possible future direction could be to add binaural inputs to the model, with the VNLL inhibition to the IC driven by contralateral octopus cells (Vater et al., 1997).

The model presented here for IC chirp-velocity sensitivity and AM tuning provides a tool for investigating the contribution of velocity sensitivity to complex sounds, such as speech responses. In speech stimuli, phase shifts due to vocal-tract filtering (Klatt, 1980) would result in frequency chirps within pitch periods. An IC model that is sensitive to chirp velocity may improve the accuracy of predictions of physiological responses to speech sounds. Given the ubiquity of such neurons in the IC (Mitchell et al., 2023), such a study would be important in elucidating the processing of speech in the midbrain.

## Figures and Tables

**Table 1 –:** Octopus-cell stage parameters and values or ranges.

Parameter Name	Value (or range)
*N*_*CF*_	3 or 4
*N*_*OCF*_	1
OCF	CF/3–3CF Hz
*d*_*CF*_ OR *d*_*OCF*_	0–2 ms
Δ_*EE*_	1 ms
Δ_*Hyp*_	2 ms
*d*_*Hyp*_	0.4 ms
*θ*	50 spikes/s

**Table 2 –:** Parameter values for example neurons

CF (kHz)	IC direction	OCF (kHz)	*d*_*CF*_ (ms)	*d*_*OCF*_ (ms)	*N*_*CF*_	*M*_*Oct*_	*M*_𝐼_	*d*_*E*_ (ms)	*d*_*I*_ (ms)	Δ_*Oct*_ (ms)	Δ_*I*_
1	Down	2.21	0	0.75	4	12	8	1	3.5	1	1
1	Up	0.90	1.20	0	4	12	16	1.4	3.4	1	1
4	Down	5.33	0.30	0	3	6	16	0.5	3.0	1	1
4	Up	2.24	1.58	0	3	6	8	1.5	4.0	1	1
8	Down	10.69	0.21	0	3	3	8	1.2	3.7	1	1
8	Up	5.51	0.93	0	3	12	8	1.5	4.5	1	1
